# Temporomandibular joint changes after orthodontic treatment with clear aligners: A scoping review

**DOI:** 10.1186/s12903-026-08414-3

**Published:** 2026-05-07

**Authors:** Ziad M. Montasser, Mona A. Montasser

**Affiliations:** 1 Faculty of Dentistry, Horus University-Egypt, New Damietta, Egypt; 2 Department of Orthodontics, Faculty of Dentistry, Mansoura University, Mansoura, 35516 Egypt

**Keywords:** Scoping review, Systematic review, Orthodontics, Clear aligners, Temporomandibular joint changes

## Abstract

**Objective:**

To review the available evidence on temporomandibular joint morphological changes after orthodontic treatment with clear aligners, as assessed using CBCT scans.

**Materials and methods:**

This review sought randomized clinical trials, controlled or comparative clinical trials, and retrospective cohort studies. The primary outcomes were condylar position and joint spaces, and the secondary outcomes were condylar dimensions and inclination, and mandibular fossa dimensions. MEDLINE (via PubMed), Scopus, Web of Science, ProQuest, and the Clinical Trials Registration and the WHO International Clinical Trials Registry Platforms were searched. The risk of bias was assessed using the ROBINS-I tool.

**Results:**

The review included four retrospective studies with high heterogeneity and risk of bias. Each study addressed a different type of malocclusions; Class I, Class II (unspecified division), Class II division 1, and Class II division 2. After clear aligner treatment in adults, no significant changes were observed in condylar position or joint spaces in Class I non-extraction and extraction cases, as well as in Class II non-extraction cases while, Class II division 2 non-extraction cases demonstrated improvement in initially posteriorly positioned condyles. A similar change was reported in skeletal Class II division 1 adolescents treated with functional clear aligners. Results suggest that growth plays a significant role in influencing condylar position and mandibular fossa dimensions.

**Conclusions:**

The evidence is derived from four studies with a serious risk of bias, therefore should be interpreted as exploratory rather than confirmatory. This review highlights a research gap that warrants further investigation through high-quality studies followed by systematic reviews.

## Introduction

 The temporomandibular joint (TMJ) is a complex structure, both anatomically and functionally, composed of the mandibular condyle (inferiorly), the squamous part of the temporal bone (superiorly), the articular disc, as well as the surrounding articular capsule and supporting ligaments [1].

The importance of the TMJ in the field of orthodontics lies primarily in two areas; the association between orthodontic treatment and temporomandibular disorders (TMDs), and the critical role the TMJ plays in growth modification, particularly in cases involving mandibular retrusion.

One of the earliest publications to establish a cause-effect relationship between occlusion and TMDs was by Costen, an otorhinolaryngology surgeon. Based on his observations of a group of edentulous patients who reported symptom improvement following the restoration of vertical dimension and occlusal contact, he concluded that occlusal problems were linked to TMDs [2]. However, current evidence indicates that the risk of occlusal features leading to the development of TMDs is low to very low. In fact, evidence supports that conditions affecting TMJ morphology or causing musculoskeletal pain are more likely to influence dental occlusion, rather than the reverse. As a result, patients may perceive changes in their occlusion due to the underlying TMDs conditions [1, 3]. As a consequence of the proposed association between occlusion and TMDs, orthodontic treatment has also been implicated as either a potential cause or a treatment for TMDs [4–6].

The TMJ has long been an important consideration in growth modification. The condylar growth potential response to the treatment of malocclusions such as Class II and Class III, as well as high or low vertical dimensions; treatment approaches and outcomes can differ significantly between growing patients and adults due to this growth potential [7].

The concept of using clear aligners for orthodontic treatment can be traced back to Kesling [8] who developed the tooth positioner, an active orthodontic retainer originally used during the retention phase to make final adjustments to minor tooth malalignments.

According to Kesling [8] the positioner can serve functions beyond final tooth positioning and retention. In theory, substantial tooth movements could be achieved by using multiple Positioners, each made with incremental adjustments to the setup as treatment advances. Although this approach was not practical at the time, it remained a potential option with potential clinical applications in the future.

After decades, the new computer-aided design & computer-aided manufacturing (CAD/CAM) technology led to the development of the Invisalign^®^ appliance for orthodontic treatment in the USA by the Align Technology incorporation in 1998. The Invisalign^®^ technique depends on developing a series of algorithmic stages to move the teeth in a series of small increments each about 0.15–0.25 mm. Therefore, the Invisalign^®^ appliance consists of a series of clear thermoplastic aligners that each is worn for 1-2-weeks [9].

Previous experimental and review studies have investigated the positional and morphological changes of the TMJ associated with conventional orthodontic treatment [10, 11]. The objective of the present scoping review was to evaluate the current evidence on temporomandibular joint morphological changes after orthodontic treatment with clear aligners, as assessed using CBCT scans.

## Materials and methods

The preferred reporting items for systematic reviews and meta-analyses extension for scoping reviews (PRISMA-ScR) [12, 13], were adopted to guide the reporting of this scoping review.

### Eligibility criteria

According to the PCC standard (participants, concept, context) the following inclusion criteria were applied:

1. *Participants*: Orthodontic patients treated with clear aligners.
2. *Concept*: Temporomandibular changes with clear aligners for orthodontic treatment.
3. *Context*: Randomized clinical trials (RCTs), controlled or comparative clinical trials (CCT), and retrospective cohort studies carried out in any setting with no language restrictions. Although scoping reviews typically include a broad range of study designs, this review focused on comparative designs to better characterize interventional effects on TMJ morphology and to assess whether sufficiently homogeneous evidence exists for future systematic reviews.

#### Exclusion criteria

Any other type of studies other than the types mentioned in the inclusion criteria. Studies that include surgical cases, cleft lip and palate cases, patients with syndromes that may affect the TMJ, and/or history of Joint fractures, juvenile idiopathic arthritis, rheumatoid arthritis, or psoriasis.

#### Information sources

Search included multiple databases namely; MEDLINE (via PubMed), Scopus, Web of Science, ProQuest Dissertations&Theses Global, Clinical Trials Registration (CTR) website https://clinicaltrials.gov/, and the WHO International Clinical Trials Registry Platform (ICTRP) https://www.who.int/clinical-trials-registry-platform. There was no language restriction or publication year limits applied. Hand searching of the references’ lists of the articles considered eligible for inclusion in the review was also done. The search was conducted on May 27, 2025, and updated approximately six months later, on November 17, 2025.

### Search strategy

(Orthodontics OR malocclusion) AND (Clear aligners OR aligners) AND (TMJ OR Temporomandibular joint OR Temporomandibular OR Condyle OR Glenoid fossa OR Fossa OR Disc) AND (Remodeling OR Positional change OR positional changes OR Change OR Changes).

### Selection of the studies eligible for inclusion

The results obtained from the databases were imported into Mendeley^®^ Desktop version 1.19.8 to identify and remove duplicate articles. After duplicate removal, articles were screened based on their titles and abstracts. The studies that passed this initial multi-level filtering process, as described, underwent a final round of screening through full-text reading and careful application.

of the inclusion criteria. The selection was conducted by one reviewer and independently verified by another reviewer; any disagreements were resolved through discussion.

### Data collection and data charting

Data from each of the included studies were extracted. The collected data included the study title, authors, and year of publication, study design, setting, participants’ characteristics, study groups, type of appliance used, treatment duration, outcomes, and outcome measures. The data were extracted by one author and double checked by another. The most relevant data were summarized in Table 1.

**Table 1:** Main characteristics of the included studies

Study	Design& setting	Patients	Intervention	Follow-up (Duration)	Imaging method	Outcome
Al-Somairi et al. [15], (2025)	**Design:** - Retrospective - Comparative study Group 1: Non-extraction (*n* = 60) Sub-group (1 a) Fixed appliance (*n* = 30) Sub-group (1 b) Clear aligners (*n* = 30) Group 2: Extraction (*n* = 60) Sub-group (2 a) Fixed appliance (*n* = 30) Sub-group (2 b) Clear aligners (*n* = 30) **Setting:** - Department of Orthodontics, School and Hospital of Stomatology, China Medical University, Shenyang 110,002, P.R. China.	- Skeletal Class I, moderate crowding - Adults (Age ˃18Years)	- Edgewise fixed appliance - Clear aligners	- Before (T0) and after treatment (T1) - Mean duration (months): Sub-group (1 a): 2.47 ± 0.73 years Sub-group (1 b): 2.21 ± 0.74 years Sub-group (2 a): 3.27 ± 0.85 years Sub-group (2 b): 2.95 ± 0.94 years	- CBCT	- Positional and morphological changes in the TMJ
Al-Worafi et al. [16], (2024)	**Design:** - Retrospective - Comparative study Group 1: Fixed appliance (*n* = 35) Group 2: Clear aligners (*n* = 35) **Setting:** - Department of Orthodontics, School and Hospital of Stomatology, China Medical University, Shenyang 110,002, P.R. China.	- Skeletal Class II &bilateral Class II molar relationship - Mild-moderate crowding in both arches - Adults (between 18years and 40years)	- Edgewise fixed appliance - Clear aligners	- Before (T0) and after treatment (T1) - Mean duration (months): Group 1: (25.69 ± 5.04) Group 2: (24.97 ± 5.17)	- CBCT	- Positional and morphological changes in the TMJ
Zheng et al. [17], (2023)	**Design:** - Retrospective - Comparative study Group 1: Fixed appliance (*n* = 25) Group 2: Clear aligners (*n* = 22) **Setting:** State Key Laboratory of Military Stomatology, National Clinical Research Center for Oral Diseases & Shaanxi Clinical Research Center for Oral Diseases, Department of Orthodontics, School of Stomatology, Air Force Medical University, Xi’an 710,032, Shaanxi, People’s Republic of China	Skeletal Class II division 2 - Adults (Age˃18years) - Mean age (years): Group 1: (22.80 ± 3.85) Group 2: (23.18 ± 3.76)	- Edgewise fixed appliance - Clear aligners	- Before (T0) and after treatment (T1) - Mean duration (months): Group 1: (23.20 ± 1.85) Group 2: (22.82 ± 2.13)	- CBCT	- Positional and morphological changes in the TMJ
Zhang et al. [18], (2024)	**Design:** - Retrospective - Comparative study Group 1: Twin-Block (*n* = 24) Group 2: Clear functional aligners (*n* = 25) **Setting:** State Key Laboratory of Oral & Maxillofacial Reconstruction and Regeneration, National Clinical Research Center for Oral Diseases & Shaanxi Clinical Research Center for Oral Diseases, Department of Orthodontics, The Third Affiliated Hospital of Air Force Medical University, Xian 710,032, Shaanxi, People’s Republic of China.	Skeletal Class II division 1 - Adolescents - Mean age (years): Group 1: (10.71 ± 0.86) Group 2: (10.96 ± 0.84)	- Twin Block functional appliance - Clear functional aligners.	- Before (T1) and after treatment (T2) - Mean duration (months): Group 1: (12.15 ± 0.99) Group 2: (11.82 ± 0.88)	- CBCT	- Positional and morphological changes in the TMJ

### Risk of bias in individual studies

The risk of bias in each of the included studies was assessed using the Risk of Bias in Non-randomized Studies - of Interventions (ROBINS-I) tool [14]. This tool is designed to evaluate the likelihood that the observed effects are attributable to the intervention itself rather than to potential sources of bias inherent in non-randomized studies. ROBINS-I comprises three main domains: pre-intervention (bias due to confounding and selection of participants into the study), at intervention (bias in classification of interventions), and post-intervention (bias due to deviations from intended interventions, missing data, measurement of outcomes, and selection of the reported result). The overall risk of bias for each study was determined based on the assessment of the individual domains. The evaluation was conducted by one reviewer and independently verified by another. Any disagreements were resolved through discussion.

### Synthesis of the results

Charting the data of the included studies revealed significant heterogeneity. Therefore, the charted data were analyzed and synthesized descriptively, consistent with the nature of a scoping review. The process involved extracting standardized data on positional and dimensional changes in the TMJ from each study, after which the findings for each outcome across all included studies were synthesized narratively. No further synthesis was made.

### Risk of bias across studies and additional analyses

Tests for publication bias were planned if a sufficient number of studies were available, with a minimum of 10 studies required to perform this analysis.

## Results

### Study selection

**Fig. 1:**
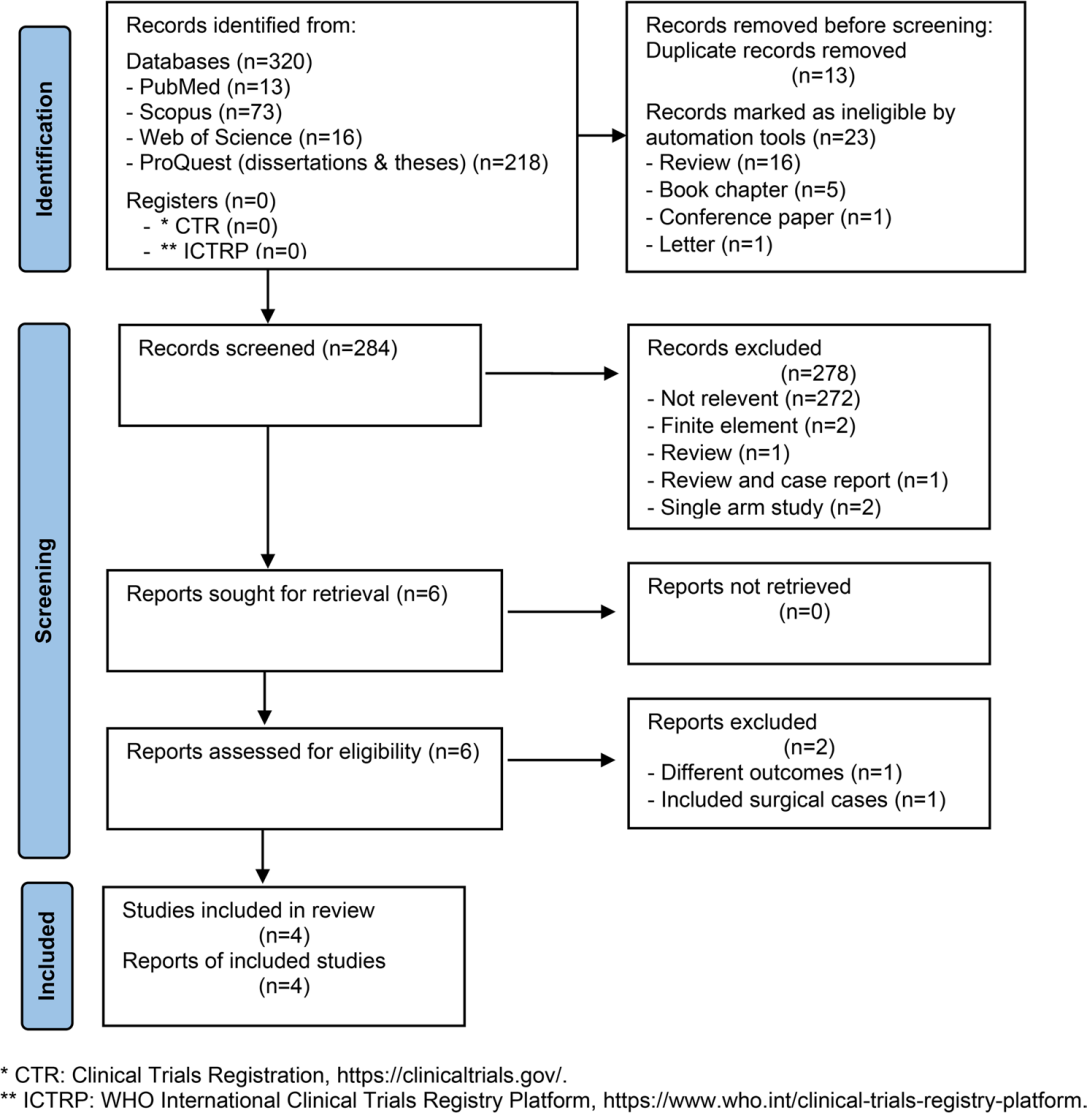
PRISMA-ScR flow diagram for the search and selection of studies for inclusion in the review

A total of 320 sources were identified through searches of databases containing published and unpublished research, distributed as follows: PubMed (*n* = 13), Scopus (*n* = 73), Web of Science (*n* = 16), and ProQuest (*n* = 218). The search of the ClinicalTrials.gov database and the WHO International Clinical Trials Registry Platform yielded no results. After removing sources using automation tools and removing duplicates, 287 sources remained. Screening the titles and abstracts for relevance to the review topic reduced this number to six. Following full-text assessment, two additional studies were excluded, resulting in four articles [15–18] suitable for inclusion, Fig. 1.

### Study characteristics

The data, presented in Table 1, showed that the four included articles were retrospective studies, published recently between July 2023 and January 2025. Each pair of the included studies originated from the same institution with overlapping research teams and similar methodologies and outcomes. Three of the studies [15–17] included adult patients and compared fixed appliances (FA) with clear aligners (CA). One of these three studies [16] further subdivided each group into extraction and non-extraction groups. Although each of the three studies addressed a different type of malocclusion, Class I, Class II (unspecified division), and Class II division 2, the malocclusion type was consistent within each individual study. The fourth study [18] involved adolescent patients with Class II division 1 malocclusion and compared treatment using a Twin-Block (TB) functional appliance versus functional clear aligners (FCA). All four studies employed cone beam computed tomography (CBCT) to assess treatment outcomes.

### Risk of bias in individual studies

Based on the seven domains of the ROBINS-I tool, the risk-of-bias assessment for each study indicated that all four included studies had a serious overall risk of bias, as summarized in Table 2. Key methodological flaws observed across the studies were primarily related to bias due to confounding, bias in selection of participants, and bias in measurement of outcomes. Although each included study investigated a different type of malocclusion and likely involved distinct, non-overlapping datasets, certain study characteristics raise important concerns. Specifically, each pair of studies originated from the same institution, involved overlapping research teams, and employed closely similar methodologies and outcome assessments. This overlap suggests the presence of institutional clustering and may limit the independence of the evidence, indicating that the four studies cannot be considered fully independent.

**Table 2:** Risk of bias assessment for non-randomized clinical trials-of interventions (ROBINS-I)

	Pre-intervention		At intervention	Post-intervention				
	Selection	Comparability	Exposure	Assessment			Reporting	
Studies	Bias due to confounding	Bias in selection of participants into the study	Bias in classification of interventions	Bias due to deviations from intended interventions	Bias due to missing data	Bias in measurement of outcomes	Bias in selection of the reported result	Overall bias
Al-Somairi et al. [15], (2025)	Moderate	Serious	Low	Low	Low	Moderate	Low	Serious
Al-Worafi et al. [16], (2024)	Moderate	Serious	Low	Low	Low	Moderate	Low	Serious
Zheng et al. [17], (2023)	Serious	Serious	Low	Low	Low	Moderate	Low	Serious
Zhang et al. [18], (2024)	Serious	Serious	Low	Low	Low	Moderate	Low	Serious

### Results of individual studies

The main changes investigated in the studies were (1) Condylar position changes through measuring anteroposterior condylar position (APCP) [15, 16], vertical condylar position (VCP) [15, 16], medio-lateral condylar position (MLCP) [15, 16], anteroposterior condylar joint position (APCJP) [15, 16], and by applying the Pullinger formula [19] in the four studies [15–18], (2) Joint spaces through measuring anterior joint space (AJS) [15–18], superior joint space (SJS) [15–18], posterior joint space (PJS) [15–18], interior joint space/medial joint space (IJS) [15–18], exterior joint space/lateral joint space (EJS) [15–18] and total mandibular joint volume (TMJV) [15, 16], (3) Condylar dimension through measuring Condylar length/internal and external diameter of the condyle (CL/IEDC) [15–18] condylar width/anterior and posterior diameter of the condyle (CW/APDC) [15–18], Height of the condyle (HC) [17, 18], Volume of the condyle (VC) [17, 18], and Surface area of the condyle (SC) [17, 18], (4) Condylar inclination through measuring anteroposterior condylar inclination (APCI) [15, 16], vertical condylar inclination (VCI) [15, 16], medial condylar inclination (MCI) [15, 16], sagittal condylar angle (SCA) [17, 18], horizontal condylar angle (HCA) [17, 18], and (5) Mandibular fossa dimensions through measuring mandibular fossa height/ mandibular fossa depth (MFH/MFD) [15–17], mandibular fossa width (MFW) [15–18].

### Synthesis of results

The data of the three studies [15–17] that focused on adult patients were grouped, while the study [18] included adolescent patients was presented separately. Adult patients [15–17] were treated using either clear CA or FA, whereas adolescents [18] received treatment with either functional CA or TB functional appliances.

### Condylar position

#### Non-extraction adult treatment

Treatment of skeletal Class I patients with CA vs. FA [15] showed that in the CA group the changes in the APCP, VCP, MCP, APCJP did not differ significantly after treatment than before treatment (*p* ≥ 0.05). In the FA group, the APCP increased from 6.11 ± 1.74 mm to 6.49 ± 1.88 mm, *p* = 0.000 and the VCP increased from 2.32 ± 1.28 mm to 2.13 ± 1.25 mm, *p* = 0.025. Intergroup comparisons showed no significant difference between the changes of the APCP, VCP, MCP, and APCJP (*p* = 0.372, *p* = 0.563, *p* = 0.681, and *p* = 0.509 respectively). Additionally, Pullinger formula [19] indicated stable posterior position (PP), increased centric position (CP), and reduced anterior position (AP) in the CA group and a slight increase in PP, a rise in CP, and a decrease in anterior position AP in the FA group.

While in skeletal Class II [16], measurements of the condylar position such as APCP, VCP, and MCP in the CA group did not change significantly (*p* ≥ 0.05), while in the FA group; the APCP decreased from 5.86 ± 2.39 mm to 5.72 ± 2.49 mm (*P* = 0.013), the VCP increased from 10.73 ± 2.49 mm to 10.90 ± 2.34 mm (*P* = 0.035), and the MCP increased from 42.83 ± 2.07 mm to 43.39 ± 2.09 mm (*P* = 0.000). Intergroup comparisons showed a significant change in the MCP which was more inward in the fixed appliance group (-0.56 ± 0.82 mm) than in the CA group (-0.12 ± 0.40 mm), *P* = 0.006. Furthermore, according to Pullinger formula [19] the condyle’s centric position in the glenoid fossa before treatment in the CA and FA groups was 45.7% and 40%, respectively and changed following treatment to 51.4% in the CA group and 31.4% in the FA group.

The study by Zheng et al. [17] on Class II division 2 adults used Pullinger’s formula [19] to assess the condyle position which showed that about 61.36% and 13.64% of the patients’ condyles were posterior to the fossa before and after CA treatment respectively. In the FA treatment, about 68% and 24% of the patients’ condyles were in the posterior portion of the articular fossa before and after treatment respectively.

### Extraction adult treatment

In Al-Somairi et al. [15] study when extractions were carried out, the condylar position in the CA group measured by the APCP, VCP, MCP, and the APCJP did not differ significantly after treatment (*p* ≥ 0.05). In the FA group there were significant increase in the APCP from 6.58 ± 1.98 mm to 7.08 ± 1.84 mm, *p* = 0.000, but the APCJP decreased from 2.39 ± 16.62 mm to -11.78 ± 16.32 mm, *p* = 0.000. Intergroup comparisons showed significant differences between the changes in the APCP and APCJP (*p* = 0.041 and *p* = 0.046 respectively), but the changes in the VCP and MCP were not different (*p* ≥ 0.05).

### Non-extraction adolescent treatment

Using Pullinger’s formula [19], Zhang et al. [18] study on growth modification of Class II division 1 found that before treatment approximately 12.0% of the condyles in the functional CA group and 12.5% of the condyles in the TB functional appliance group were in the posterior portion of the articular fossa. After treatment, the condyles in both the groups were in the neutral position of the articular fossa.

### Joint spaces

#### Non-extraction adult treatment

Similar results were found in the studies by Al-Somairi et al. [15] and Al-Worafi et al. [16] where joint spaces showed no significant change in either group. Intergroup comparisons showed no significant difference between the changes in the AJS, SJS, PJS, MJS, and VTJS (*p* ≥ 0.05). Also, the study by Zheng et al. [17], only the SJS was significantly different between the two groups as it increased in the CA group and decreased in the FA group by 0.70 ± 0.40 mm and 0.04 ± 0.15 mm respectively, *P* < 0.001.

### Extraction adult treatment

In the extraction groups of Al-Somairi et al. [15] study, intra-group comparison showed no significant change in any of the joint spaces in the CA group, however, there was significant increase in APJS from 2.57 ± 0.56 to 2.85 ± 0.67 mm and a significant decrease in the PJS from 2.68 ± 0.56 to 2.36 ± 0.47 mm in the FA group. Intergroup comparisons showed no significant difference between the changes in the AJS, SJS, PJS, MJS, and TMJV (*p* ≥ 0.05).

### Non-extraction adolescent treatment

Functional treatment of Class II division 1 with CA increased significantly the SJS from 2.93 ± 0.17 mm to 3.01 ± 0.22 mm, (*P* = 0.024), but the TB appliance group did not show similar change. The changes in the anterior, superior, posterior, medial, and lateral joint spaces occurred in the two groups were not different (*p* ≥ 0.05) [18].

### Condylar dimension

#### Non-extraction adult treatment

In non-extraction adult treatment of skeletal Class I patients, the increase in CL (0.07 ± 0.70 mm) and CW (0.11 ± 0.50 mm) in the CA group were similar to the increase in CL (0.16 ± 0.47 mm) and CW (0.14 ± 0.64 mm) in the FA group [15]. In non-extraction adult treatment of skeletal Class II patients condylar dimensions did not change significantly after treatment in either group and the changes were not significantly different between the two groups [16]. In Class II division 2, all of the condylar dimensional measurements; IEDC, APDC, HC, SC, and VC increased significantly in both groups except the increase in the HC in the CA group that increased from 7.77 ± 0.75 mm to 7.83 ± 0.65 mm, *p* = 0.181 [17].

### Extraction adult treatment

Extractions did not change CL or CW significantly in either CA or FA groups after treatment [15].

### Non-extraction adolescent treatment

Functional treatment of adolescent Class II division 1 malocclusion resulted in significant changes in condylar dimensions. The mean increases in CH were 2.38 ± 0.90 mm and 1.97 ± 0.92 mm (*p* = 0.028), VC were 60.55 ± 139.78 mm³ and 83.98 ± 137.46 mm³ (*p* = 0.405), and SC were 112.49 ± 124.34 mm³ and 118.23 ± 110.73 mm³ (*p* = 0.825) in the CA and TB groups, respectively. The mean increase in IECD was 0.90 ± 1.47 mm and 1.39 ± 1.03 mm (*p* = 0.060), while the APCD increased by 0.86 ± 0.86 mm and 1.79 ± 1.0 mm (*p* = 0.001) in the CA and TB groups, respectively [18].

## Condylar inclination

### Non-extraction adult treatment

In Class I malocclusion, the only significant changes in condylar inclination were in the FA group where the VCI decreased significantly from 58.15 to 56.16°, (*p* = 0.022), and the MCI increased significantly from 8.90 to 10.07°, (*p* = 0.034) after treatment. However, these changes were not significantly different compared to the changes in the CA group [15]. In Class II malocclusion, the only significant change in the condylar inclination was the change in the APCI in the FA group from 74.27 ± 5.50° to 74.14 ± 4.94°, *P* = 0.008). However, this change was not significant compared to the change in the CA group.^16^ In Class II division 2, condylar inclination changes in the CA and FA groups were negligible and statistically insignificant [17].

### Extraction adult treatment

Condylar inclination did not change significantly in either the CA or the FA groups [15].

### Non-extraction adolescent treatment

Condylar inclination changes in functional CA and TB functional appliance groups were negligible and statistically insignificant [18].

### Mandibular fossa dimensions

#### Non-extraction adult treatment

In Class I and Class II, mandibular fossa dimensional changes were insignificant in the two groups [15, 16]. In Class II division 2, dimensions of the glenoid fossa changed in the two groups; in the CA group the MFW increased from 24.83 ± 2.09 mm to 24.85 ± 1.01 mm, (*p* = 0.760) and from 25.66 ± 2.49 mm to 26.09 ± 2.47 mm, (*p* = 0.001) in the FA group. In the CA group the depth (MFD).

increased from 11.36 ± 0.80 mm to 11.70 ± 0.70 mm, (*p* = 0.001) while increased from 11.21 ± 1.06 mm to 11.22 ± 1.0 mm, (*p* = 0.808) in the FA group. Intergroup comparison showed that the changes were significantly different between the two groups [17].

### Extraction adult treatment

Also, mandibular fossa dimensions measurements did not change significantly in either group [15].

### Non-extraction adolescent treatment

After functional treatment of adolescent Class II division 1 malocclusion, mandibular fossa dimensions increased in the two groups; in the CA group the MFD increased from 29.12 ± 1.30 mm to 29.45 ± 1.33 mm, (*p* = 0.149) and from 28.94 ± 0.94 mm to 29.41 ± 1.41 mm, (*p* = 0.075) in the TB group. In the CA group the MFW increased from 13.36 ± 0.73 mm to 14.77 ± 1.12 mm, (*p* = 0.001) while increased from 13.64 ± 0.78 mm to 15.37 ± 1.01 mm, (*p* = 0.001) in the TB group. These changes were comparable between the two groups [18].

### Risk of bias across studies and additional analyses

Given the limited number of included studies, formal tests for publication bias were not performed as such analyses require a larger sample to yield meaningful results.

## Discussion

Observed TMJ changes likely reflect adaptive responses to malocclusion treatment. Specific treatment effects, such as mandibular unlocking in Class II Division 2, leveling a deep curve of Spee in Class II Division 1, leveling and alignment adjustments, and closing extraction spaces may lead to redistribution of occlusal forces [20, 21] and altered loading of the TMJ [22]. In contrast to the well-established and extensively characterized force systems of FA, the biomechanics of clear aligners remain less predictable and continue to evolve. This is reflected in the reported discrepancy, around 50%, between predicted and achieved tooth movements [23]. Such differences are largely attributed to the distinct nature of force delivery in clear aligners, where force application and transmission are governed by the material properties of the thermoplastic appliance as well as its geometric configuration [24].

In the current review, non-extraction treatment of adult patients with skeletal Class I malocclusion, moderate crowding, and an overjet of approximately 3–4 mm using CA might have negligible effects on condylar position and joint spaces [15]. In contrast, treatment with FA might significantly increase the APCP and decrease the APCJP, although no significant changes were observed in the joint spaces. However, when the CA and FA groups were compared, the differences in condylar position and joint spaces were not statistically significant. These outcomes may be influenced by the type of malocclusion and the use of a non-extraction treatment protocol. In the same study [15], the CA extraction group showed no significant differences in condylar position or joint spaces after treatment, similar to the findings in the non-extraction group. Conversely, the FA extraction group demonstrated significant changes in APCP, APCJP, APJS, and PJS.

Previous investigations have evaluated condylar position in Class II subjects in comparison with those with Class I malocclusion or normal occlusion. The study by Sharma et al. [25] utilized CBCT scans to compare condylar positions in untreated healthy adult patients with skeletal Class I and Class II malocclusions. The findings revealed that condyles in Class II patients were positioned more anteriorly, as indicated by reduced anterior joint spaces and increased posterior joint spaces. Additionally, a lower condylar position was observed with an increased superior joint space [25]. Similarly, the study by Rivero-Millán et al. [26] reported that, compared with normal occlusion, the condyles in Class II division 1 patients were positioned more anteriorly. These positional differences suggest that condylar characteristics associated with Class II malocclusion may undergo changes following orthodontic correction.

In the current review, the study by Al-Worafi et al. [16] evaluated non-extraction treatment of adult patients with skeletal Class II malocclusion and mild to moderate crowding using CA with sequential molar distalization. The study demonstrated no significant changes in condylar position or joint spaces. The reporting of this study did not specify the type of Class II malocclusion, whether it was division 1, division 2, or a combination, complicating the interpretation of the results. A single-arm retrospective study by Al-Tayar et al. [27] evaluated the effects of CA sequential distalization from molars to incisors in adult patients with Class II dental relationships. Although significant improvements were observed in molar distalization and molar relationships, no statistically significant changes were detected in the dimensional or positional parameters of the TMJ, even with concurrent changes in vertical dimension. In contrast, in the same study [16], the FA group demonstrated significant changes in condylar position, including a decrease in APCP and increases in both VCP and MCP, while joint spaces remained unchanged.

Class II division 2 malocclusion differs from Class II division 1 in several key morphological characteristics. Rivero-Millán et al. [26] evaluated condylar position in healthy individuals aged 16 years or older with Class II division 1 and Class II division 2 malocclusions, compared with those with Class I occlusion. The findings indicated that condyles were positioned more posteriorly in individuals with Class II division 2 malocclusion compared with Class I subjects. In the study included in the current review by Zheng et al. [17], approximately 61.4% of patients in the CA group had condyles positioned posteriorly within the glenoid fossa prior to treatment. Similarly, in the FA group, about 68% of condyles were located posteriorly. The study reported a decrease in anterior joint space and an increase in posterior joint space in both groups, suggesting a forward displacement of the condyle toward the center of the glenoid fossa after treatment. These findings suggest that, irrespective of appliance type, changes in the sagittal position of the condyle occurred following Class II division 2 correction and unlocking of the mandible. Notably, only the CA group exhibited an increase in superior joint space and in the depth of the glenoid fossa, whereas such changes were not observed in the FA group. The authors interpreted these findings as evidence of adaptive condylar remodeling during orthodontic treatment, particularly in patients treated with clear aligners.

The effects of growth modification in adolescents treated with functional CA or TB appliances on condylar positional changes have been an area of interest. Using Pullinger’s formula [19], the study by Zhang et al. [18] reported improvements in condylar position in both CA and TB groups: condyles shifted from a pre-treatment posterior position within the articular fossa to a post-treatment more neutral position. Similarly, a study by Wu et al. [28] investigated mandibular protrusive condylar trajectory in adolescents with skeletal Class II Division 1 malocclusion undergoing treatment with functional CA. The results showed that these adolescents exhibited a greater protrusive range compared with those with Class I malocclusion, as indicated by significant increases in anteroposterior and spatial condylar displacements after approximately eight months of treatment.

When evaluating changes in condylar inclination in the current review, Al-Somairi et al. [15] reported significant changes in VCI) and MCI in the FA non-extraction group. However, the changes in condylar inclination were not significantly different when comparing CA and FA treatments in either the extraction or non-extraction subgroups. Thus, although FA treatment was associated with more pronounced changes in condylar position, inclination, and joint spaces, these changes were not significantly different from those observed with CA treatment. Similarly, studies evaluating adult Class II [16] and adult Class II division 2 malocclusions [17] reported negligible changes in condylar inclination following treatment with either CA or FA. In adolescents with Class II division 1 malocclusion [18], changes in condylar inclination were also comparable between patients treated with functional CA and those treated with the TB appliance.

Condylar and mandibular fossa changes in adults are expected to differ from those observed in adolescents due to the influence of growth. In adult Class I patients [15], both extraction and non-extraction groups showed stable condylar and mandibular fossa dimensions following treatment with either modality. Similarly, in adult Class II patients [16], these structures remained largely unchanged regardless of the treatment approach. These findings may be explained by the limited growth potential in adult patients. In Class II division 2 cases [17], although no statistically significant differences were observed in the depth of the glenoid fossa among the studied malocclusions, both the CA and FA groups demonstrated a comparable and significant increase in condylar-related dimensions. This increase may be related to the age of the participants, as the included patients were 16 years or older, and to the effect of mandibular unlocking following the proclination of the maxillary incisors during treatment.

Functional appliances primarily exert their effects through growth modification mechanisms. Consistent with this principle, the only study in the current review that included adolescents [18], which evaluated the treatment of Class II division 1 malocclusion using both functional CA and the TB appliance, reported significant post-treatment increases in all condylar dimension measurements in both groups. Although the changes in CV, CS, and IEDC did not differ significantly between the groups, intergroup comparisons revealed significantly greater changes in CH and APDC. Additionally, mandibular fossa width and depth increased significantly in both groups, with no significant difference between the two treatment modalities [18]. Similarly, the systematic review and meta-analysis by Zybutz et al. [11] examined TMJ changes in growing Class II patients treated with functional orthodontic appliances. The authors reported limited and low-quality evidence suggesting a short-term association between functional appliance therapy and morphological changes in the TMJ. These changes included increased condylar width, decreased anterior joint space, increased superior and posterior joint spaces, and vertical displacement of the glenoid fossa. However, the clinical relevance of these findings remains uncertain, highlighting the need for further high-quality, long-term studies.

The clinical significance of the statistically significant CBCT-detected changes reported in this review should be interpreted with caution. Although measurements taken from CBCT scans are generally considered accurate for craniofacial measurements [29, 30], small changes of less than 1 mm have been suggested to be clinically insignificant, as these differences may be attributed to measurement error rather than a true physical change [31, 32]. Several parameters associated with the CBCT technique, such as voxel size, spatial resolution, segmentation thresholds, and reconstruction algorithms, may affect the accuracy of the measurements [30, 33]. Notably, the absence of clinical TMD outcome measures (e.g., pain, joint sounds, or functional limitation) in all included studies limits the clinical interpretability of the imaging findings. Clinical assessments would be helpful in complementing the findings obtained from CBCT scans.

The main limitations of this review arise from concerns regarding the internal and external validity of the included studies. All included studies were assessed as having a high risk of bias. All were retrospective in design which is frequently criticized for its susceptibility to various sources of bias. In particular, the risk of bias in the included studies was mainly related to selection bias, resulting from the absence of randomization and the inclusion of only successfully treated cases, which may have led to an overestimation of treatment results. Additional methodological limitations included the lack of allocation concealment and blinding, as well as the presence of potential confounding factors, such as imbalances in sex distribution and variations in specifics of treatment mechanics among the study samples. Furthermore, in the growth modification study, the absence of a control group makes it difficult to distinguish between true treatment effects and growth-related changes. These methodological limitations may substantially influence the reported treatment outcomes and should therefore be carefully considered when interpreting the findings of the included studies and, consequently, the conclusions of this review. On the other hand, at the level of the body of evidence, the external validity of the findings also raises concerns. Only four studies were included, with each pair originating from the same institution and involving overlapping research team members, suggesting evident institutional clustering and raising concerns regarding the independence of the evidence. In addition, substantial heterogeneity was observed across the studies. Therefore, the generalizability of the findings is likely limited.

## Conclusions

- This scoping review focused on temporomandibular joint morphological changes after clear aligner orthodontic treatment assessed from CBCT scans.
- After CA treatment, condylar position and TMJ spaces appear to remain largely unchanged in adult Class I (extraction and non-extraction) and Class II non-extraction cases, forward condylar displacement and changes in joint spaces may be seen in Class II division 2 non-extraction, and growth-related effects on condylar and mandibular fossa dimensions following functional CA are also suggested.
- The evidence is derived from a small number of studies with a serious risk of bias, thereby limiting its clinical relevance. The findings should therefore be interpreted as exploratory rather than confirmatory.
- A research gap is highlighted that warrants further investigation through high-quality research followed by systematic reviews.

## Data Availability

No datasets were generated or analysed during the current study.

## Abbreviations

CA: Clear aligners
FCA: Functional clear aligners
FA: Fixed appliance
TB: Twin-Block
TMJ: Temporomandibular joint
TMDs: Temporomandibular disorders
CAD/CAM: Computer-aided design & computer-aided manufacturing
CBCT: Cone beam computed tomography
AJS: Anterior joint space
PJS: Posterior joint space
SJS: Superior joint space
MJS: Medial joint space
VTJS: Volumetric total joint space
AP: Anterior position
PP: Posterior position
CP: Centric position
APCP: Anteroposterior condylar position
VCP: Vertical condylar position
MCP: Medial condylar position
LCP: Lateral condylar position
MLCP: Mediolateral condylar position
APCJP: Anteroposterior condylar joint position
APCI: Anteroposterior condylar inclination
VCI: Vertical condylar inclination
MCI: Medial condylar inclination
CH: Condylar height
CW: Condylar width
VC: Volume of the condyle
SC: Surface area of the condyle
MFH: Mandibular fossa height
MFW: Mandibular fossa width
WGF: Width of glenoid fossa
DGF: Depth of glenoid fossa
IEDC: Internal and external diameters of the condyle
APDC: Anterior and posterior diameter of the condyle
